# Low-Dose Sevoflurane Promotes Hippocampal Neurogenesis and Facilitates the Development of Dentate Gyrus-Dependent Learning in Neonatal Rats

**DOI:** 10.1177/1759091415575845

**Published:** 2015-04-06

**Authors:** Chong Chen, Feng-Yan Shen, Xuan Zhao, Tao Zhou, Dao-Jie Xu, Zhi-Ru Wang, Ying-Wei Wang

**Affiliations:** 1 Department of Anesthesiology and Intensive Care Medicine, Xinhua Hospital, College of Medicine, Shanghai Jiaotong University, China; 2 Institute of Brain Functional Genomics, East China Normal University, Shanghai, China; 3 Graduate School, Institute of Science and Technology Austria, Klosterneuburg, Austria

**Keywords:** dentate gyrus, neonate, neurogenesis, pattern separation, sevoflurane, spatial learning

## Abstract

Huge body of evidences demonstrated that volatile anesthetics affect the hippocampal neurogenesis and neurocognitive functions, and most of them showed impairment at anesthetic dose. Here, we investigated the effect of low dose (1.8%) sevoflurane on hippocampal neurogenesis and dentate gyrus-dependent learning. Neonatal rats at postnatal day 4 to 6 (P4–6) were treated with 1.8% sevoflurane for 6 hours. Neurogenesis was quantified by bromodeoxyuridine labeling and electrophysiology recording. Four and seven weeks after treatment, the Morris water maze and contextual-fear discrimination learning tests were performed to determine the influence on spatial learning and pattern separation. A 6-hour treatment with 1.8% sevoflurane promoted hippocampal neurogenesis and increased the survival of newborn cells and the proportion of immature granular cells in the dentate gyrus of neonatal rats. Sevoflurane-treated rats performed better during the training days of the Morris water maze test and in contextual-fear discrimination learning test. These results suggest that a subanesthetic dose of sevoflurane promotes hippocampal neurogenesis in neonatal rats and facilitates their performance in dentate gyrus-dependent learning tasks.

## Introduction

In the developing brain, hippocampal neurogenesis, the process by which new bron neurons are generated from neural stem and progenitor cells in the dentate gyrus (DG), is a common phenomenon. Over the past years, there have been considerable evidence to demonstrate that neurogenesis can be influenced by volatile anesthetics (Engelhard et al., 2007; Stratmann et al., 2009; Zhu et al., 2010; Culley et al., 2011; Fang et al., 2012; Erasso et al., 2013; Lin et al., 2013; Nie et al., 2013; Zhang et al., 2013).

The prevailing view suggests that, in the developing brain, many volatile anesthetics induce widespread neurodegeneration or impairment of neurogenesis in parallel with long-term learning or memory deficits at anesthetic doses (Jevtovic-Todorovic et al., 2003; Stratmann et al., 2009; Zhu et al., 2010; Fang et al., 2012). However, a recent finding indicates a treatment-intensity-dependent dual effect of isoflurane on proliferation and differentiation in human neuroprogenitor cells, that is, short-term exposure to low isoflurane concentrations promoted proliferation and differentiation, whereas prolonged exposure to high isoflurane concentrations induced cell damage and inhibited proliferation (Zhao et al., 2013). This dual effect was also observed *in vivo* with altered behavior performance in rats. Fetal exposure to low concentrations of isoflurane improved spatial memory performance (Li et al., 2007) but induced memory and learning deficits with exposure to higher isoflurane concentrations (Kong et al., 2012). Therefore, it is reasonable to ask whether this is a common phenomenon of volatile anesthetics and whether other volatile anesthetics, such as sevoflurane, have similar effects. Indeed, a recent report indicates that brief (1 hour) exposure to sevoflurane at clinical concentrations enhances proliferation of cultured neural stem cells, but a 6-hour exposure suppresses proliferation and induces apoptosis (Nie et al., 2013). However, except for these neurotoxic effects of anesthetic doses on cultured cells, the *in vivo* effects of low sevoflurane concentrations on the developing brain are yet to be determined. Here, we therefore aimed to investigate the effect of a subanesthetic dose (1.8%) of sevoflurane, an anesthetic which is widely used in pediatric anesthesia and shows less neurotoxicity than isoflurane (Liang et al., 2010), on hippocampal neurogenesis in neonatal rats. Furthermore, because of clues indicating that hippocampal neurogenesis plays important roles in learning and memory formation (Shors et al., 2001; Aimone et al., 2006; Bruel-Jungerman et al., 2007; Deng et al., 2010; Sahay et al., 2011; Suarez-Pereira et al., 2014), we also investigated the hippocampal DG-dependent learning ability after exposure to low sevoflurane concentrations.

## Materials and Methods

### Animals

All experiments were conducted with the approval of the Institutional Animal Care and Use Committee at Shanghai Jiaotong University and East China Normal University. Sprague-Dawley dams with litters containing male pups were raised in the Animal Center of Key Laboratory of Brain Genomics at East China Normal University. To control for litter variability, several pups were used from each litter in each treatment condition.

### Anesthetic Methods

Rats were randomly allocated to either control or anesthesia exposure groups on postnatal day 4 to 6 (P4–6). Rats in the anesthesia group were placed in a thermostated anesthetic chamber, and body temperature was maintained at 37℃ with a heating blanket. Sevoflurane (Fushimi-machi, Osaka, Japan) anesthesia was induced using a concentration of 3.5% and 1.5 L/min 70% oxygen + 30% air over 3 min and maintained at 1.8% for 6 hours. For the control group, rats were exposed to 70% oxygen + 30% air for 6 hours in the thermostated anesthetic chamber. Anesthesia gas monitoring was performed using an analyzer (Drager, Germany) with the sensor inside the anesthetic chamber. To ensure adequate oxygenation and respiration, arterial blood gas determination was performed by cardiac puncture at 2 hours and at the end of the anesthesia. Arterial blood gases were measured with a portable clinical analyzer (Radiometer Instruments, Denmark). Serum corticosterone levels of rats in both groups were assayed at the termination of anesthesia to exclude the influence of stress response. Mixed venous blood was obtained from rapid decapitation, and corticosterone levels were determined by ELISA (Crystal Chem., Inc).

### Bromodeoxyuridine Injections and Immunohistochemistry

For the bromodeoxyuridine (BrdU) injections, we followed the methods described by Tozuka et al (2005). To investigate the specific effects of 1.8% sevoflurane on cellular proliferation, 24 hours after anesthesia, rats were intraperitoneally (i.p.) administered a single 300 mg/kg BrdU injection. One day later, they were perfused, and their brains were processed for immunohistochemistry. To investigate the effect of 1.8% sevoflurane on the survival of newborn cells, rats received a single injection of BrdU 24 hours before their exposure to sevoflurane. Four weeks (28 days) after the BrdU injection, the animals were perfused, and their brains were processed for immunohistochemistry. For perfusions, all rats were deeply anesthetized with sodium pentobarbital (80 mg/kg i.p.) and transcardially perfused with 4% paraformaldehyde in 0.1 M phosphate buffer.

Brain sections, 40 µm thick, were cut with a frozen section machine (Leica Instruments, Germany). For BrdU and DAPI double-labeling immunofluorescence, brain sections were treated with 10 mM sodium citrate buffer (pH = 6) at 92℃ to 98℃ for 15 min and incubated with blocking solution (5% goat serum, 3% Triton, and 3% BSA in 0.1 M PBS) for 120 min at room temperature. Following rinsing, the tissues were incubated overnight with mouse anti-BrdU (1:250, Millipore, USA). Alexa 488 secondary antibodies conjugated to the appropriate species were applied for 2 hours (Vector, USA). Following rinsing, the sections were incubated with DAPI (1:200, Beyotinme, China) for 30 min. Double-label immunofluorescence was detected by a fluorescence microscope (Olympus, Japan). During the photography for one marker, the strength of the laser was held constant as were all other image analysis settings. Quantification was conducted in a blind manner. Every 10th section throughout the hippocampus was processed for BrdU immunohistochemistry. Image J software (Version 1.48 V, National Institutes of Health, USA) was used to merge the graphs with different markers. BrdU/DAPI-positive cells were manually counted by another experimenter with the help of the Cell Counter function of Image J.

### Brain Slice Preparation and Patch Clamp Recordings

Rats, aged P14 to 16, were used for electrophysiological recordings. After being deeply anesthetized using sodium pentobarbital (80 mg/kg i.p.), Sprague-Dawley rats were decapitated, and their brains were rapidly removed and placed in an ice-cold high sucrose artificial cerebrospinal fluid (ACSF) solution (in mM: 252 sucrose, 3 KCl, 2 MgSO_4_, 24 NaHCO_3_, 1.25 NaH_2_PO_4_, 1.2 CaCl_2_, and 10 glucose) bubbled with carbogen (95% O_2_ + 5% CO_2_). Two min later, the entire hippocampal formation was dissected and then cut into 400-µm slices using a vibrating microtome (VT-1200; Leica instruments). Slicing was performed in ice-cold high sucrose ACSF and then moved to the normal ACSF (in mM: 119 NaCl, 2.5 KCl, 2.5 CaCl, 1.3 MgSO_4_, 11 D-Glucose, 1 NaH_2_PO_4_, and 26.2 NaHCO_3_) for incubation at room temperature. At least 2 hours later, the slices were transferred to a recording chamber for recordings.

For patch clamp recording, brain slices were continuously perfused with normal ACSF via a gravity-fed perfusion system and maintained at 30℃. The DG region was identified by contrast microscopy (Olympus, Japan). Whole-cell patch electrodes were pulled using P-97 (Sutter Instruments, USA) from borosilicate glass (1.5 mm outer diameter and 1.1 mm inner diameter; Sutter Instruments) to resistances ranging from 5 to7 MΩ. The intracellular solution contained (in mM) 140 K-gluconate, 11 EGTA, 2 MgCl_2_, 1 CaCl_2_, 10 HEPES, and 2 K_2_ATP. Recordings were performed using an Axon 700B patch clamp amplifier (Axon Instruments, USA). The amplified signals were digitized at 5 kHz using a Digidata 1440 attached to Clampfit 10.1 (Axon instruments, USA). Recordings having steady series resistances of <30 MΩ (typically 18–26 MΩ) were the only ones included in the data pool. After a successful patch, a 50-pA stop current was given to examine the following parameters: cell input impedance, spike threshold, and spike amplitude. We divided the granular cell layer (GCL) of the DG into inner and outer halves according to the description by Scobie et al. (2009). As the immature neurons concentrated in the inner half of the GCL, the recordings were performed mainly in this location.

### Morris Water Maze

The behavioral tests were performed in a quiet, light-controlled room. Before the start of the water maze tests, rats were acclimatized to the environment of the test room and the researchers for three days. Rats from both groups were placed in a 120-cm diameter plastic pool filled with clean water, which was then dyed black. The temperature of the water was maintained at 23℃ ± 1℃. Visual cues were placed around the edges of the pool to help rats locate the submerged platform. For each trial, rats were gently placed into the pool at one of four different locations and allowed 60 s to locate the platform submerged 2 cm below the surface of the water. The platform was randomly located in one of four assigned positions and was kept constant for each individual rat during the acquisition tests. The rats were guided to the platform if they were unable to find it. Once on the platform, the rats were allowed to remain there for 30 s. At the end of each trial, the rats were taken out from the pool, towel dried, and then returned to their home cage. The acquisition task lasted for five consecutive days, with one session each day consisting of four trials with an interval of 30 min each session. Each trial was videotaped, and the swim paths were tracked using Water Maze software (Coulbourn Instruments, USA). The distance traveled, latency to reach the platform, and swim speed were calculated for each trial and then averaged over each daily session. A probe test was conducted 24 hours after the end of the training phase. During the test, the platform was removed and the animals were allowed to swim freely in the pool for 60 s. The percentage of time that animals spent in each of the four quarters were calculated and used for assessment of the long-term memory for the platform location.

### Open Field Test

The open field test is a standard test of both anxiety and locomotor behavior (Scobie et al., 2009). The open field (Tru Scan, USA) is an arena 1 m in diameter that consists of a simple square enclosure equipped with infrared detectors to track animal movement in the horizontal and vertical planes. Rats were put in open field for 30 min. The total distance traveled, the proportion of the path in the center, and the proportion of time spent in the center for individual rats in both groups were recorded and analyzed by TRU SCAN2.1 software.

### One-trial Contextual Fear Conditioning

One-trial contextual fear conditioning was performed 10 days after the water maze test. Conditioning was conducted on one side of a shuttle box with clear Plexiglas walls and a stainless steel grid as a floor. On the days of testing, rats were brought out from the animal house and allowed to habituate for 1 hour outside the testing room before starting the experiment. Each rat’s behavior was recorded by a digital video camera mounted above the conditioning chamber. FreezeFrame and FreezeView software (Precision, USA) were used for recording and analyzing freezing behavior, respectively. The one-trial contextual fear conditioning protocol entails the delivery of a single 2-s foot shock of 0.8 mA at 185 s after placement of the rats in the training context. Fifteen seconds after the termination of the foot shock, rats were taken out and returned to their home cages. For the training context A, the house lights were turned on, stainless steel grids were exposed, and a mild lemon scent was used as an olfactory cue. Ethanol (75%) was used to clean the grids and chamber walls in between runs. For the distinct context C, the stainless steel grid floor and two of the chamber walls were replaced with plastic panels. The house lights were switched off, and the chamber door was left ajar during testing.

### Contextual Fear-discrimination Learning Test

The procedure was adapted from previous studies (Scobie et al., 2009; Sahay et al., 2011). Following one-trial contextual fear conditioning, we conducted contextual fear-discrimination learning to test the animals’ ability to distinguish between two similar contexts: conditions that are most likely to recruit the DG (McHugh et al., 2007), specifically the shock-associated training context A and the similar (nonshock) context B. Context B shared many features with context A, including an exposed stainless steel grid floor and roof. The similar context differed from the training context in that two plastic panels were used to cover the chamber walls, the house lights were switched off, and the chamber door was left ajar during testing. A mild pineapple scent was used as an olfactory cue, and a nonalcoholic antiseptic was used to clean the grids between runs. Rats were brought into the testing room in buckets by the same experimenter who had handled them in the one-trial contextual fear conditioning learning test. For discrimination learning, rats were exposed to the training context in which they received a single 2-s foot shock of 0.8 mA at 185 s after placement in the chamber. Fifteen seconds after the termination of the foot shock, rats were taken out and returned to their home cages. After 1 hour, rats were placed in the similar context, in which they were left for 180 s and were never shocked. The measurement of the freezing levels in both the training context (180 s preshock) and the similar context each day allowed for the assessment of discrimination between the two contexts until there was a significant difference between the control and anesthesia exposure groups.

### Statistical Analyses

Data are shown as *M* ± *SEM*. Statistical analyses were performed using Sigma Stat. 3.5 software. Statistical significance was assessed by a paired *t* test, unpaired *t* test, or analysis of variance (ANOVA) followed by Fisher’s post hoc tests, where appropriate.

## Results

### Sevoflurane Monitoring, Blood Gases, and Corticosterone Levels

The concentration of sevoflurane in the closed chamber was monitored during anesthesia. After 3.5% sevoflurane induction, the concentration was kept at 1.8% during the whole period (data not shown). According to a previous study, the minimum alveolar concentration of sevoflurane for neonatal rats is over 3% (Orliaguet et al., 2001); thus, 1.8% sevoflurane is a subanesthetic or low concentration for the pups. No pups died during anesthesia. Blood gas parameters and corticosterone levels were used to exclude the influence of hypercapnia and the stress response. Compared with the control group, there were no significant changes in pH (*n* = 4 per group, *p* = .896, one-way ANOVA followed by Normality Test), pCO_2_ (*n* = 4 per group, *p* = .948, one-way ANOVA followed by Normality Test), pO_2_ (*n* = 4 per group, *p* = .594, one-way ANOVA followed by Normality Test), or corticosterone levels (*n* = 9 per group, *p* = .226, unpaired *t* test) in the sevoflurane treatment group. (Table 1).

**Table 1.** The Effects of Low-Dose Sevoflurane on Blood Gases and Corticosterone Levels.

Parameter	Control	Sevoflurane (2 h)	Sevoflurane (6 h)
pH	7.38 ± 0.05	7.37 ± 0.04	7.36 ± 0.04
Pco_2_^a^ (mmHg)	36.00 ± 8.28	37.60 ± 10.87	38.00 ± 7.35
Po_2_^b^ (mmHg)	135.70 ± 9.21	132.60 ± 10.31	133.50 ± 10.34
Cort^c^ (ng/L)	114.84 ± 5.62	–	129.87 ± 10.54

a Pco_2_ = partial pressure carbon dioxide.

b Po_2_ = partial pressure oxygen.

c Cort = corticosterone.

### Low-Dose Sevoflurane Promotes Neuronal Proliferation and Increases the Survival of Newborn Cells in DG

**Figure 1.**
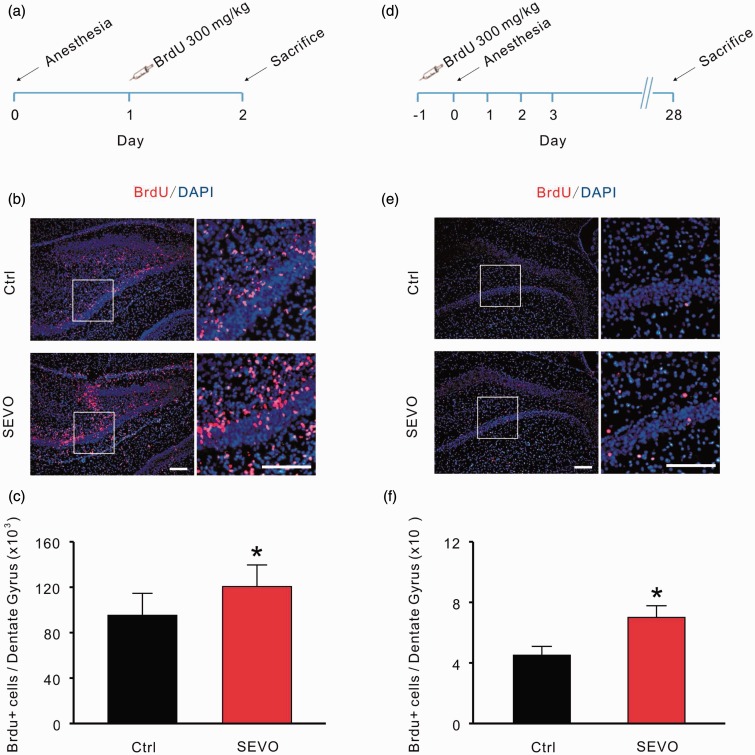
Low-dose sevoflurane promotes neuronal proliferation and increases the survival of newborn cells in the dentate gyrus. (a) Protocol of BrdU inject ions for testing the proliferation of progenitor cells in the dentate gyrus. (b) Positive signals of cells for both the proliferative marker BrdU (red) and DAPI (blue). Scale bar represents 100 µm. (c) Summary data for the experiment are presented in (b). (d) Protocol of BrdU injection for testing the survival of newborn cells. (e) Positive signals of cells for both BrdU and DAPI. Scale bar represents 100 µm. (f) Summary data for the experiment are presented in (e) control group, sevoflurane group; *p < .05. Ctrl = control group; SEVO = sevoflurane group.

To investigate the effect of 1.8% sevoflurane on the proliferation of progenitor cells in the DG, BrdU (300 mg/kg i.p.) was injected into animals in both groups 24 hours after 1.8% sevoflurane treatment, and the animals were sacrificed for immunostaining 24 hours later (Figure 1(a)). From the results, we can see that many cells were labeled by BrdU in each section from both groups, which means that hippocampal neurogenesis is active in neonatal rats (Figure 1(b)). The statistical results show that the number of BrdU/DAPI double-positive cells in the sevoflurane group is significantly larger than the control group (control: 95,207 ± 19,432 vs. sevoflurane: 120,564 ± 19,036; *n* = 6 per group; *p* = .027, unpaired *t* test; Figure 1(c)).

Previous studies have suggested that it would take 3 to 4 weeks for newborn cells to integrate functionally into the hippocampal circuit (Zhao et al., 2006; Ge et al., 2007; Toni et al., 2007). To evaluate the surviving cells that have successfully joined into the circuit, we labeled dividing cells with a single BrdU injection and then treated neonatal rats with 1.8% sevoflurane for 6 hours (Figure 1(d)). Rats were sacrificed 28 days after BrdU injection, and the total number of newborn cells was counted by double immunostaining with anti-BrdU and DAPI (Figure 1(e)). Data from immunofluorescence staining demonstrated that treatment with 1.8% sevoflurane significantly increased the number of surviving newborn cells in the DG (control: 4,505 ± 584 vs. sevoflurane: 7,000 ± 775; *n* = 6 per group; *p* = .026, unpaired *t* test; Figure 1(f)).

### Low-Dose Sevoflurane Increases the Proportion of Immature Granular Cells in DG

**Figure 2.**
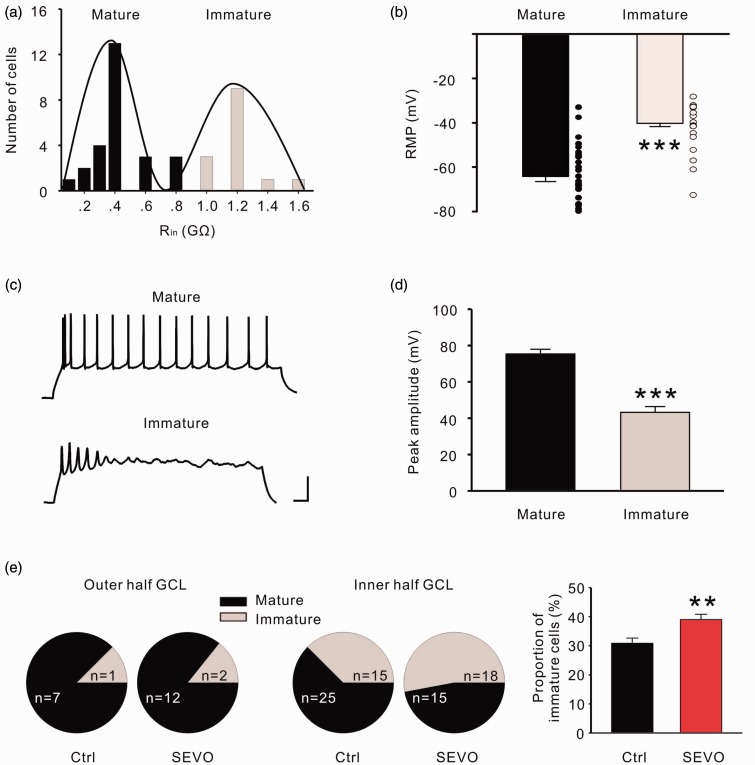
Low dose of sevoflurane promotes neuronal proliferation and increases the survival of newborn cells in the dentate gyrus. (a) Input resistance (*R*_in_) distribution of two type granular cells in the dentate gyrus. Continuous curves represent the sum of two Gaussian functions fitted to the histogram. (b) The resting membrane potential of mature granular cells is more negative than in immature cells. (c) Action potential properties of mature and immature granular cells in response to the current step. Scale bars represent 50 mV and 50 ms. (d) Amplitudes of the first action potential from mature and immature granular cells. (e) The proportion of immature granular cells in outer half (left), inner half (middle), and total (right) of granular cell layer. **p < .01, ***p < .001. Ctrl = control group; SEVO = sevoflurane group.

After being generated in the subgranular zone, it will take approximately 1 week for newborn granular cells to migrate into the inner half of the GCL (Altman and Bayer, 1990). We used electrophysiological patch clamp recording to discriminate the immature and mature granular cells, which show distinct differences in input resistance (*R*_in_), resting membrane potential, and firing style (Liu et al., 1996; Schmidt-Hieber et al., 2004). We divided the GCL into the inner half and outer half and then blindly performed whole cell recording bilaterally. According to the distribution of *R*_in_, most cells fell into two categories, with mean *R*_in_ values of 178 ± 51 MΩ and 1.3 ± 0.1 GΩ (*n* = 40 cells from four animals; Figure 2(a)). The resting membrane potential of granular cells with the smaller *R*_in_ is more negative than the larger *R*_in_ cells (−64.2 ± 2.3 mV vs. − 40.2 ± 1.6 mV; Figure 2(b)). When inward current injection (50 pA, 1 s), unlike mature granular cells that fire trains of regular action potentials, immature granular cells generated only a few action potential per stimulus (Figure 2(c)), and the action potential amplitude of mature granular cells was higher than the immature ones (74.5 ± 2.2 mV vs. 42.7 ± 2.9 mV; Figure 2(d)). When analyzing the outer half of the GCL, we found that rats in both groups showed a comparable proportion of immature granular cells (Figure 2(e) left). However, in the inner half of the GCL, the percentage of immature granular cells is up to 52.9% in the sevoflurane group, while it is only 37.5% in the control group (Figure 2(e) middle). The total proportion of immature granular cells in the sevoflurane group (*n* = 7) was greater than the control group (*n* = 7; *p* = .008, unpaired *t* test; Figure 2(e) right).

The results above indicate that a 6 hours treatment with 1.8% sevoflurane increased the proportion of immature granular cells in the DG of neonatal rats by promoting hippocampal neurogenesis and the survival of newborn cells.

### Low-Dose Sevoflurane Facilitates the Rats’ Performance in the Morris Water Maze Test

The results from BrdU labeling suggested that 1.8% sevoflurane promotes hippocampal neurogenesis and increases the survival of newborn cells in the DG. Many studies on the physiological function of hippocampal neurogenesis have shown that newborn cells are important for spatial learning memory (Kee et al., 2007), particularly for the acquisition of new information (Kempermann and Gage, 2002). Therefore, we chose the Morris water maze test to check the effects of 1.8% sevoflurane on hippocampal DG-dependent spatial learning and memory.

**Figure 3.**
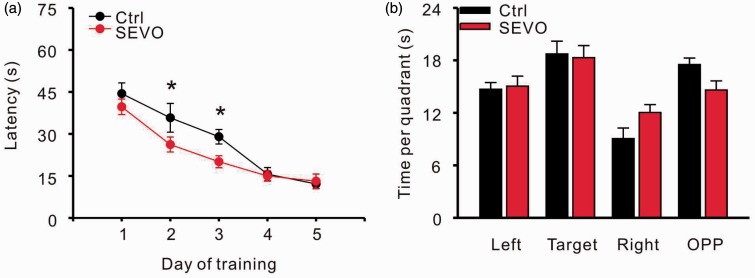
Low dose of sevoflurane facilitates performance in the Morris water maze test. (a) Time to find the platform on five training days. (b) The probe test after five days of training. *p < .05. Ctrl = control group; SEVO = sevoflurane group.

Four weeks after anesthesia, juvenile rats in the control and sevoflurane groups were trained to learn the location of the submerged platform in the water maze. Rats that underwent a 6-hour 1.8% sevoflurane exposure spent significant shorter time than controls to find the hiding platform on learning [*n* = 9 in control and 12 in sevoflurane group; two-way repeated ANOVA, (treatment) *F* = 6.486, *p* = .012, (treatment × day) *F* = 1.400, *p* = .240; Figure 3(a)], especially on day 2 (control: 35.77 ± 5.12 s vs. sevoflurane: 26.20 ± 2.68 s; *p* = .019) and day 3 (control: 29.00 ± 2.59 vs. sevoflurane: 20.07 ± 2.14; *p* = .028). After five days of training, rats in both groups spent a similar amount of time to find the hidden platform, and there were no significant differences in their performance in the probe test (Figure 3(b)).

**Figure 4.**
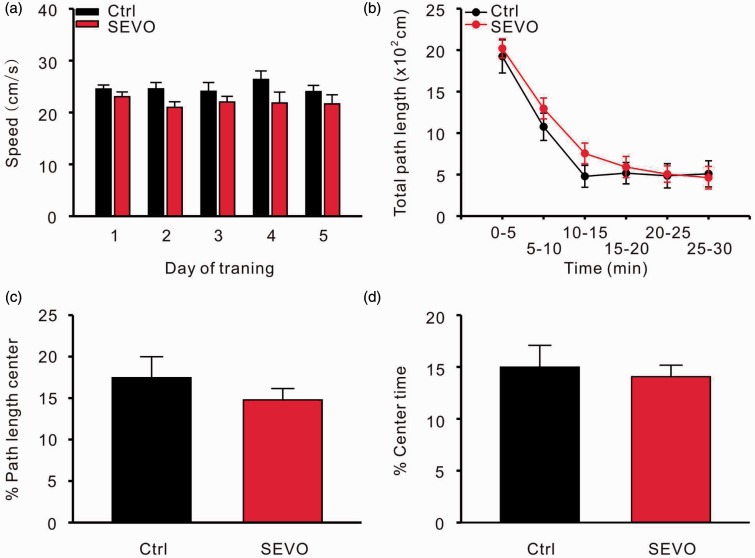
Low dose of sevoflurane does not influence motor system and anxiety level of rats. (a) Swimming speeds during the training days for the Morris water maze. Open field test for total path length (b), path length in center (c), and time in center (d). Ctrl = control group; SEVO = sevoflurane group.

To exclude the influence of a low-dose sevoflurane treatment on the motor system and emotion, we analyzed the swimming velocity of rats in both groups during the training days. An open field test was also performed to investigate the anxiety level of the animals. The velocities of the rats from both groups were not significantly different during the five training days [two-way repeated ANOVA, (treatment) *F* = 3.528, *p* = .087, (treatment × day) *F* = 0.709, *p* = .590; Figure 4(a)]. A 6-hour exposure to 1.8% sevoflurane had no effect on the total path length [*n* = 10 in control and 12 in sevoflurane group; two-way repeated ANOVA, (treatment) *F* = 1.781, *p* = .185, (treatment × time) *F* = 0.384, *p* = .859; Figure 4(b)], proportion of path length in the inner arena (control: 16.8 ± 2.3% vs. sevoflurane: 12.6 ± 1.9%; *p* = .184, unpaired *t* test; Figure 4(c)), or the time spent in the inner arena (control: 14.9 ± 2.1% vs. sevoflurane: 11.2 ± 2.5%, *p* = .286, unpaired *t* test; Figure 4(d)) during the 30 min observation in the open field test.

### Low-Dose Sevoflurane Facilitates Performance in Pattern Separation

The hippocampal DG is thought to transform similar experiences or events into discrete, nonoverlapping representations, which is a process known as pattern separation (Treves et al., 2008). Hippocampal neurogenesis, especially the birth of young granular cells, is critical for pattern separation (Scobie et al., 2009; Sahay et al., 2011; Nakashiba et al., 2012). According to our results, 4 weeks after treatment, there were more BrdU positive cells in the DG of rats from the sevoflurane group compared with the control group. Therefore, we chose a contextual fear-discrimination learning test to investigate the effect of 1.8% sevoflurane on pattern separation.

**Figure 5.**
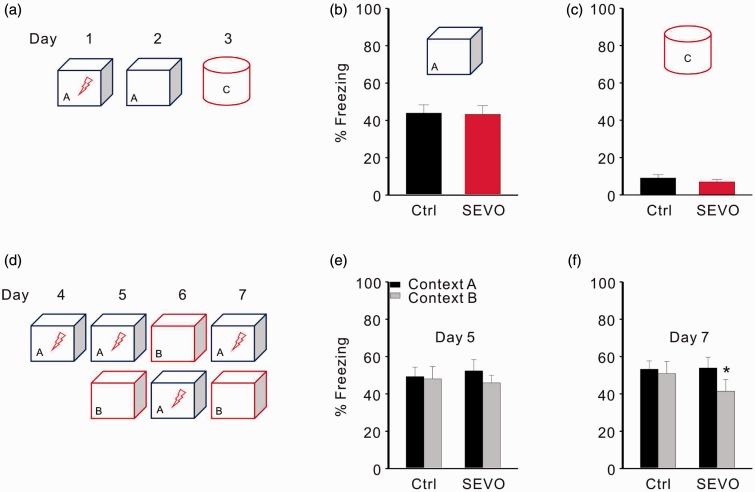
Low dose of sevoflurane facilitates pattern separation in rats. (a) Protocol for a single trial contextual fear-conditioning test. (b) Freezing level for the training context A. (c) Freezing level for the distinct context C. (d) Protocol for the contextual fear-discrimination learning test. (e) Freezing level for training context A and similar context B on day 5. (f) Freezing level for training context A and similar context B on day 7. *p < .05. Ctrl = control group; SEVO = sevoflurane group.

To test whether increased hippocampal neurogenesis influences rapid contextual encoding, we subjected rats from both groups to a single trial contextual fear-conditioning test (Figure 5(a)). Rats from the control and sevoflurane groups showed similar levels of freezing during a contextual test 24 hours after being trained in context A, suggesting that both groups acquired and retained contextual fear memory equally well (control: 49.70 ± 14.44% vs. sevoflurane: 42.22 ± 11.33%; *n* = 9 in control and 12 in sevoflurane group; *p* = .921, unpaired *t* test; Figure 5(b)). Like control rats, those in the sevoflurane group showed negligible levels of freezing behavior in distinct context C, which had few features in accordance with training context A (Figure 5(c)). These results indicate that fear conditioning in both groups was specific to the training context and that increased hippocampal neurogenesis does not affect the ability of an animal to distinguish between two markedly different contexts.

We next investigated whether the increased newborn cells in the DG affect a form of learning that requires an animal to distinguish between similar contexts A and B (Figure 5(d)). On day 5, when rats in both groups were initially exposed to context B, they showed comparable levels of freezing as in context A, suggesting that context B shared enough features with context A to elicit a generalization of contextual fear in both groups (Figure 5(e)). An analysis of freezing behavior over multiple days for each group in both contexts showed that rats in the sevoflurane group learned to distinguish differences between the two contexts on day 7 (context A: 53.9 ± 5.7% vs. context B: 43.1 ± 6.5%; *p* = .027, paired *t* test), but control rates were still unable to distinguish two contexts at this time point. Furthermore, the freezing levels in the training context (context A) were not significantly different between the two groups (control: 53.2 ± 4.48 vs. sevoflurane: 53.9 ± 5.7; *p* = .930, unpaired *t* test; Figure 5(f)).

Both behavior tests showed that the rats with sevoflurane treatment learned faster than control animals, indicating that a 6-hour treatment with 1.8% sevoflurane facilitated the DG-dependent learning abilities.

## Discussion

Sevoflurane is one of the most commonly used general anesthetics, particularly in pediatric anesthesia for its hemodynamic stability, sweet smell, lack of respiratory irritation, and fast onset of action. Here we found that a 6-hour subanesthetic dose (1.8%) of sevoflurane treatment promoted hippocampal neurogenesis in neonatal rats. To our knowledge, this is the first study to demonstrate that a single treatment with low concentration sevoflurane promoted the survival of newborn cells and increased the proportion of immature granular cells in the DG of neonatal rats. This differs from previous studies that indicated a suppression by sevoflurane of neurogenesis and an increase in neurotoxicity both in stem and progenitor cells and in 7-day-old rats, when sevoflurane was used at anesthetic doses (Fang et al., 2012; Nie et al., 2013; Zhang et al., 2013). Moreover, we also found that rats that underwent a 6-hour treatment with 1.8% sevoflurane performed better in the acquisition stage of the Morris water maze test and in the contextual discrimination fear learning test, tasks that have been shown to be affected by hippocampal neurogenesis (Kee et al., 2007; Sahay et al., 2011; Nakashiba et al., 2012). Our findings are consistent with previous reports which demonstrated that subanesthetic doses of sevoflurane provide neuroprotection in hypoxic-ischemic animal models (Luo et al., 2008; Chen et al., 2014; Ren et al., 2014). High concentrations of sevoflurane have also been reported to promote neurogenesis in the DG after cerebral ischemia (Engelhard et al., 2007), perhaps indicating that neurons in the ischemic brain respond differently to sevoflurane than in normal conditions.

The dual effects of neuroprotection and neurotoxicity by general volatile anesthetics have been demonstrated by an extensive body of work both *in vitro* (Zheng and Zuo, 2003; Bickler et al., 2005; Xie et al., 2007; Culley et al., 2011) and *in vivo* (Culley et al., 2004; Zhao and Zuo, 2004; Kitano et al., 2007; Dong et al., 2009). These complicated results suggest that different parameters such as dose, timing, and exposure duration of volatile anesthetics are critical to the final outcomes and reflect the complexity of volatile anesthetics’ effects on the central nervous system. Overall, our research and other work discussed suggest that the dual effects likely arise, at least in part, from cellular responses that are dependent on anesthetic dosage or treatment duration.

Sevoflurane not only affects progenitor cells in the DG but also affects the hippocampal CA1 pyramidal neurons (Liu et al., 2014) and may have additional effects on other brain regions (Lee et al., 2013). Therefore here in this study, we cannot exclude that the behvioral effects of 1.8% sevoflurane may involve influences on the function of other brain regions, such as neurogenesis in subventricular zone (SVZ), or even some potential neurotoxic effects. For example, one study has indicated that a 6-hour exposure to approximately 0.6 minimum alveolar concentration of sevoflurane increased neocortical cell death and apoptosis in neonatal mice (Istaphanous et al., 2011).

Compared with adult or aged animals, neonates have been shown to be more sensitive to sevoflurane treatment (Callaway et al., 2012; Cao et al., 2012; Fang et al., 2012; Shih et al., 2012). In addition, neurogenesis and neurocognitive function of animals at different ages were differentially affected by isoflurane treatment with impairment in P7 rats but improvement in P60 rats (Stratmann et al., 2009). In the context of our experimental conditions, whether and how 1.8% sevoflurane affects hippocampal neurogenesis and learning performance in adult rats awaits further investigation.

In the developing brain, neurotransmitters such as GABA and glutamate have been shown to modulate the proliferation and differentiation of neural progenitor cells (LoTurco et al., 1995; Haydar et al., 2000; Nguyen et al., 2003). It is well known that in mature neurons GABA acts as an inhibitory neurotransmitter; however, in immature neurons, due to the delayed development of the K^+^-Cl^–^coupled co-transporter (KCC2), activation of GABA_A_ receptors mediates a depolarizing shift of membrane potential (Ben-Ari et al., 1989; Leinekugel et al., 1995; Ben-Ari, 2002). GABA-induced depolarization or excitation of neural progenitor cells or newborn neurons has been shown to promote neuronal differentiation and synaptic integration (LoTurco et al., 1995; Tozuka et al., 2005; Ge et al., 2006). Compared to the adult animals, the depolarizing GABAergic network activity is stronger in postnatal animals, correlating with a promotional effect on hippocampal neurogenesis (Overstreet-Wadiche et al., 2006). As a GABA_A_ receptor agonist and also a modulator (Rudolph and Antkowiak, 2004; Garcia et al., 2010), sevoflurane was shown to potentiate depolarizing GABA_A_ currents in neonatal rats (Lim et al., 2014). Thus, it is tempting to speculate that low dose sevoflurane may promote hippocampal neurogenesis through enhancement of depolarizing GABAergic neuronal activity.

Previous studies have suggested that newborn neurons play an important role in spatial learning and memory formation (Ramirez-Amaya et al., 2006; Kee et al., 2007), and increasing evidence suggests that newborn neurons might be involved in hippocampal functions that are particularly dependent on the DG, such as pattern separation (Sahay et al., 2011; Nakashiba et al., 2012). In the current study, we selected the Morris water maze and contextual discrimination fear learning test to evaluate spatial learning memory and pattern separation, respectively. What we found is that rats that underwent 1.8% sevoflurane treatment performed better in the pattern separation test. In comparison, in the water maze test, the improved performance of rats in the sevoflurane-treated group is observed only in the acquisition phase but not in the probe test. This result is consistent with a previous study which indicated that the number of DG newborn neurons correlate with the animals’ performance during the acquisition phase of the water maze task, but not the probe test performance (Kempermann and Gage, 2002).

In summary, our findings demonstrate that exposure to low concentration sevoflurane promotes hippocampal neurogenesis in neonatal rats, which may in turn mediate the improvement of rats’ performance in DG-dependent learning tasks. These findings provide new insights into the function of sevoflurane and point to potential new approaches for reversing hippocampal neurogenesis deficits.
